# MopA, the Mn Oxidizing Protein From *Erythrobacter* sp. SD-21, Requires Heme and NAD^+^ for Mn(II) Oxidation

**DOI:** 10.3389/fmicb.2018.02671

**Published:** 2018-11-13

**Authors:** Michael Medina, Antonia Rizo, David Dinh, Briana Chau, Moussa Omidvar, Andrew Juarez, Julia Ngo, Hope A. Johnson

**Affiliations:** Department of Biological Science, Center for Applied Biotechnology Studies, California State University Fullerton, Fullerton, CA, United States

**Keywords:** manganese, Mn, heme, peroxidase cyclooxygenase, lactoperoxidase, NAD^+^, PQQ, MopA

## Abstract

Bacterial manganese (Mn) oxidation is catalyzed by a diverse group of microbes and can affect the fate of other elements in the environment. Yet, we understand little about the enzymes that catalyze this reaction. The Mn oxidizing protein MopA, from *Erythrobacter* sp. strain SD-21, is a heme peroxidase capable of Mn(II) oxidation. Unlike Mn oxidizing multicopper oxidase enzymes, an understanding of MopA is very limited. Sequence analysis indicates that MopA contains an N-terminal heme peroxidase domain and a C-terminal calcium binding domain. Heterologous expression and nickel affinity chromatography purification of the N-terminal peroxidase domain (MopA-hp) from *Erythrobacter* sp. strain SD-21 led to partial purification. MopA-hp is a heme binding protein that requires heme, NAD^+^, and calcium (Ca^2+^) for activity. Mn oxidation is also stimulated by the presence of pyrroloquinoline quinone. MopA-hp has a *K*_M_ for Mn(II) of 154 ± 46 μM and *k*_cat_ = 1.6 min^−1^. Although oxygen requiring MopA-hp is homologous to peroxidases based on sequence, addition of hydrogen peroxide and hydrogen peroxide scavengers had little effect on Mn oxidation, suggesting this is not the oxidizing agent. These studies provide insight into the mechanism by which MopA oxidizes Mn.

## Introduction

Manganese (Mn) oxidizing bacteria and fungi catalyze the oxidation of Mn(II) to Mn(III, IV) leading to the formation of Mn(III, IV) oxides, which can affect the fate of Mn, iron, other metals, carbon, and sulfur due to the adsorptive and reactive properties of the Mn(III, IV) oxides (Tebo et al., 2004, 2005; Miyata et al., 2007; Gao et al., 2015; Butterfield et al., 2016). Thus, Mn oxidizing microorganisms have a profound effect on the biogeochemical cycling of many elements. The importance of Mn oxidizing bacteria and fungi are well-appreciated, as they can significantly increase the rate of Mn oxidation over abiotic processes (Tebo et al., 2004; Morgan, 2005). Yet, the diverse molecular mechanisms of Mn oxidation, especially in bacteria, remain to be resolved.

Bacteria and fungi use both direct and indirect means to oxidize Mn. Fungi oxidize Mn as part of lignin degradation using both Mn peroxidases and multicopper oxidases (Makela et al., 2016). In addition, multicopper oxidases and superoxide production are involved in the accumulation of Mn oxides by fungi (Miyata et al., 2004; Hansel et al., 2012; Tang et al., 2013). Direct Mn-oxidation in bacteria occurs by two different enzymes – multicopper oxidases and peroxidase cyclooxygenases (also referred to as animal heme peroxidases). Multicopper oxidases have been identified in several clades of Mn-oxidizing bacteria, including *Pseudomonas* sp., *Leptothrix* sp., and *Pediomicrobium* sp. (Brouwers et al., 2000; Ridge et al., 2007; Geszvain et al., 2013); but the best-characterized enzyme among these is the MnxGEF protein complex derived from *Bacillus* sp. PL-12 (Butterfield et al., 2013; Tao et al., 2015; Butterfield and Tebo, 2017; Soldatova et al., 2017a,b; Tao et al., 2017a,b). The heterologously expressed and purified MnxGEF complex can perform the two electron oxidation of Mn(II) to Mn(IV) by funneling electrons through mononuclear Type I and trinuclear copper centers to dioxygen. Recent studies on the Mnx complex (Soldatova et al., 2017a,b) indicate a unique activation of the enzyme by Mn(II) is required. Binding of a second Mn(II) forms a hydroxide bridged Mn(II)-OH-Mn(II) complex that reduces the high Mn(III)/Mn(II) potential (*E*′ = 1.5 V) to enable electron transfer to the low potential Type I Cu site.

Peroxidase cyclooxygenases capable of Mn(II) oxidation have been identified in the alphaproteobacteria *Aurantimonas manganoxydans*, *Erythrobacter* sp. SD-21 (Anderson et al., 2009), and *Roseobacter* sp. Azwk-3b (Andeer et al., 2015). *Pseudomonas putida* GB-1, which can oxidize Mn(II) using multicopper oxidases, also contains a peroxidase cyclooxygenase that can catalyze Mn oxidation (Geszvain et al., 2016). These enzymes, referred to as MopA, produce Mn(III), identified through pyrophosphate (PP) trapping in *Erythrobacter* sp. SD-21 (Johnson and Tebo, 2008). The Mn(III)-PP is not further oxidized to Mn(IV) oxides, indicating a one electron enzyme catalyzed oxidation reaction. The mechanism of Mn(II) oxidation catalyzed by MopA enzymes is not well characterized and may occur directly (Anderson et al., 2009; Nakama et al., 2014) or indirectly (Andeer et al., 2015). Peroxidase cylcooxygenases, such as myeloperoxidase and lactoperoxidase (Furtmuller et al., 2006), have been shown to oxidize compounds with high-redox potentials such as chloride and bromide to produce hypochlorous and hypobromous acids, which are used in the immune response. These and other heme peroxidases oxidize the substrate by utilizing the strong oxidative power of hydrogen peroxide to initially oxidize the heme, which is then reduced by the substrate. Despite this key role for hydrogen peroxide in heme peroxidases, the role of hydrogen peroxide in the Mn oxidizing bacterial heme peroxidases is not quite as clear. The enzyme identified in *Aurantiomonas manganoxydans* has been shown to be stimulated by hydrogen peroxide (Anderson et al., 2009), which is consistent with a typical peroxidase mechanism. In contrast, Mn oxidizing activity in cell-free extracts of *Erythrobacter* sp. SD-21 has not been shown to be stimulated by the addition of hydrogen peroxide (Johnson and Tebo, 2008), and *Escherichia coli* cell-free extracts containing recombinant MopA from *Erythrobacter* sp. SD-21 (Nakama et al., 2014) showed no increase in Mn oxidation upon addition of hydrogen peroxide, with higher concentrations of hydrogen peroxide having an inhibitory effect (Nakama et al., 2014).

Indirect bacterial Mn oxidation, catalyzed by a peroxidase cyclooxygenase, has been described in the marine alphaproteobacterium, *Roseobacter* sp. AzwK-3b. This strain enzymatically produces superoxide, which then reacts with Mn(II) to form Mn(III) and hydrogen peroxide (Learman et al., 2011). This activity has been studied in cell-free filtrates and is thought to be an oscillating peroxidase, which alternates between superoxide production, which oxidizes the Mn(II), and peroxidase/catalase activity, which removes the hydrogen peroxide product in order to prevent Mn(III) reduction and allow oxidized Mn to accumulate (Andeer et al., 2015). Hydrogen peroxide addition did not affect activity (Learman et al., 2011).

The peroxidase cyclooxygenase superfamily of heme peroxidases, containing the environmentally relevant (Anderson et al., 2011) MopA-type Mn oxidizing proteins, are distinct from the peroxidase catalase peroxidases, which contain the well-studied Mn peroxidases important for fungal lignin degradation (Anderson et al., 2009; Zamocky et al., 2015). The peroxidase cyclooxygenase and peroxidase catalase superfamilies are unrelated in sequence and structure, but have similar mechanisms of activity, employing a heme cofactor and hydrogen peroxide. The MopA-type proteins, members of the subfamily of long peroxicins (Zamocky et al., 2015), are large proteins containing the peroxidase domain, PERCAL calcium binding motifs (Santamaria-Hernando et al., 2012), and a hemolysin type calcium binding domain (Anderson et al., 2009; Zamocky et al., 2015). The domains are often repeated as found in *Aurantimonas manganoxydans* (Anderson et al., 2009), *Roseobacter* sp. AzwK-3b (Andeer et al., 2015), and *Pseudomonas putida* GB-1 (Geszvain et al., 2016). In contrast, *Erythrobacter* sp. SD-21 has one peroxidase domain and one calcium binding domain (Anderson et al., 2009), making this protein a good model for investigating these novel enzymes. Because of difficulties expressing and purifying the full length 2138 amino acid protein (Johnson and Tebo, 2008; Nakama et al., 2014), a truncated Mn oxidizing protein, MopA-hp, containing only the heme peroxidase domain was used in this study. Partial purification and characterization of the heterologously expressed MopA-hp from *Erythrobacter* sp. SD-21 indicates a heme protein that requires NAD^+^ but not hydrogen peroxide for Mn oxidation, suggesting a novel mechanism may be involved in Mn oxidation.

## Materials and Methods

### Cloning of MopA-hp From *Erythrobacter* sp. SD-21

MopA-hp was cloned into pSpeedET using PIPES cloning as described previously (Klock et al., 2007), but consisted of amino acids 1-969, which led to slightly higher activity than the construct described in Nakama et al. (2014) (amino acids 1-929). Amplification of MopA-hp was performed using the following primers: 5′-CTGTACTTCCAGGGCATGGCCGTCAAACTCAACAAGC-3′ and 5′-AATTAAGTCGCGTTAGGTGTCATCACCGGCCGTGCCG-3′. Amplification reactions were performed with high fidelity PFU Turbo (Agilent) according to the manufacturer’s instructions. The product of the *mopA-hp* amplification reaction was gel purified prior to transformation with the amplified pSpeedET vector into *E. coli* HK100. The constructed plasmid was confirmed by sequencing the *mopA-hp* gene and then transformed into *E. coli* Rosetta^TM^ 2 (Novagen) for protein expression.

### Expression and Purification of MopA-hp

*Escherichia coli* Rosetta^TM^ 2 pSpeedET *mopA-hp* was grown overnight at 37°C in LB media containing 60 μg/ml kanamycin and 30 μg/ml chloramphenicol. Overnight cultures were transferred into fresh media with the appropriate antibiotics and grown at 37°C to an OD_600_ = 0.5 when protein expression was induced with 0.02% L-arabinose. Four hours following induction, the cultures were harvested by centrifugation at 9100 ×*g* for 15 min at 4°C. Cell pellets were resuspended in 50 mM HEPES pH 8, 50 mM NaCl, 10 mM imidazole (equilibration buffer) at 4°C. Cells were lysed by three passages through a French press cell at 16,000 psi. The cell lysate was clarified by centrifugation at 18,500 ×*g* for 20 min, 4°C.

MopA-hp was purified by nickel affinity chromatography (NAC). For gravity-driven purification, 1 ml Ni-NTA agarose beads (Qiagen) were incubated with 3 ml clarified cell lysate for 1 h at 4°C. The beads were settled into a column, and the unbound material was removed. The column was washed with five column volumes (CVs) of equilibration buffer followed by three CVs 50 mM HEPES pH 8, 300 mM NaCl, 20 mM imidazole, and 10% glycerol buffer. MopA-hp was eluted in five CVs 50 mM HEPES pH 8, 300 mM NaCl, 40 mM imidazole, and 10% glycerol. For pump-driven purification (FPLC), an IMAC column (GE Healthcare) was charged with nickel and then equilibrated with equilibration buffer, washed with 5 CVs of the equilibration buffer and then eluted over 20 CVs with a gradient of imidazole to a final concentration of 90 mM imidazole and 100 mM NaCl. Fractions with the band of interest and activity were pooled. The protein was dialyzed against 20 mM HEPES pH 8, 10% glycerol using a MWCO 10,000 dialysis cassette at 4°C for 48 h or desalted with 20 mM HEPES pH 8, 100 mM NaCl, 10% glycerol using a PD-10 (GE Healthcare) column. The protein was then concentrated with an Amicon Ultra centrifugal filter unit (10,000 Da) to a final concentration of 0.5 to 2 mg/ml.

Untransformed Rosetta^TM^ 2 cells were grown and subjected to purification in the same manner as above except kanamycin was omitted for growth. These samples are referred to as Rosetta^TM^ 2 “purified” protein controls. For chloride-free preparations, sulfate salts were substituted for all chloride salts. For anaerobically prepared NAC purified MopA-hp, all purification steps were performed in a Coy anaerobic glove bag with an atmosphere of 98% nitrogen, 2% hydrogen.

### Protein Mass Spectrometry

Protein eluted from gravity-driven affinity chromatography was sent for trypsin digestion and liquid chromatography tandem mass spectrometry (LC-MS/MS) for bulk analysis at the Donald Danforth Plant Science Center. Scaffold (version 4.3.4, Proteome Software Inc.) was used for analysis.

### Mn Oxidation Activity Assay

Mn oxidation assays contained approximately 0.1 to 0.2 mg/ml purified protein, 10 mM calcium chloride, 1 mM Mn chloride, 0.1–0.5 mM NAD^+^, 10 μM pyrroloquinoline quinone (PQQ), 0.5–1 μM heme in 50 mM HEPES pH 8, 59 mM sodium chloride. The assay mixture was incubated for 12–24 h at room temperature on an orbital shaker at 250 rpm unless otherwise indicated. Oxidized Mn accumulates linearly during this time frame. Oxidized Mn was determined with the leucoberbelin blue assay as described previously (Tebo et al., 2007). All assays were performed in triplicate. For the chloride-free assay, sulfate salts were substituted for chloride salts, except for the hemin. Heat-denatured protein was prepared by incubating the sample in boiling water for 5 min. For kinetic experiments, LBB assays were conducted hourly for 8 h after the start of the reaction and the triplicate assays contained 0.7 μM heme, 0.5 mM NAD^+^. Kinetic parameters were calculated by fitting data to the Michaelis–Menten equation using KaleidaGraph graphing software (Synergy, Inc.). Superoxide dismutase (SOD) (Millipore, Cu-Zn SOD from bovine erythrocytes) and catalase were added at 2.5–10 μM and 200 U, respectively, as indicated. For dimethylthiourea (DMTU) addition experiments, 1 mM was used in the assay. When indicated, hydrogen peroxide was added to the assay at concentrations ranging from (1–100 μM). Anaerobic assays were conducted in a Coy anaerobic chamber with 98% nitrogen, 2% hydrogen atmosphere.

### Heme Binding Assay

Hemin (Sigma-Aldrich) was dissolved in 100% dimethyl sulfoxide (DMSO), and the concentration was determined from A_405_ values using an extinction coefficient of 1.83 × 10^5^ L mol^−1^ cm^−1^ (Collier et al., 1979). Protein content was quantified by A_280_ values using an extinction coefficient of 8.824 × 10^4^ L mol^−1^ cm^−1^ (Gasteiger et al., 2003). The absorbance spectrum from 350 to 600 nm was measured on a Cary 50 spectrophotometer (Agilent). Heme binding was identified by the shift in absorbance in the Soret region. For determination of heme-protein stoichiometry, increasing amounts of heme were added to quartz cuvettes containing either 3 μM MopA-hp or buffer only. The change in absorbance at 411 nm was calculated from the difference spectrum between the two cuvettes (Kulmacz and Lands, 1984).

### SDS–PAGE

Sodium dodecyl sulfate polyacrylamide gel electrophoresis (SDS–PAGE) was performed as previously described (Laemmli, 1970) with 12% acrylamide separating and 4% acrylamide stacking gels. Proteins were stained in a 0.05% Coomassie Brilliant Blue R-250 solution in 10% acetic acid, 40% ethanol. Gels were destained in 10% acetic acid, 10% ethanol.

### NADH Detection

NADH was detected at discrete time points by measuring the A_340_ of the Mn oxidizing assay mixture following filtration (0.2 μ). In the absence of Mn, A_340_ was measured continuously. Measurements were performed on a Cary 50 spectrophotometer (Agilent) or a plate reader (BioTex). The concentration of NADH was determined using the extinction coefficient 6220 M^−1^ cm^−1^ (Dawson et al., 1986). NADH was also determined in a coupled assay in which pyruvate and lactate dehydrogenase were added to the Mn oxidation assay. In the presence of NADH, pyruvate would be reduced to lactate by lactate dehydrogenase. Lactate was then measured by a lactate oxidase-based colorimetric assay kit (Cell Biolabs, Inc). Rosetta 2^TM^ “purified” protein was used as a control for background lactate formation. For abiotic reaction assays, NADH oxidation was measured at 340 nm, freshly prepared Mn(III) acetate was added at 200 μM, PQQ at 10 μM, and NADH at 50–200 μM.

## Results

### Partial Purification of MopA-hp

The full length (2138 amino acids) Mn oxidizing peroxidase cyclooxygenase MopA protein from *Erythrobacter* sp. SD21 consists of an N-terminal heme peroxidase domain and a C-terminal calcium-binding domain based on sequence analysis. All experiments described here are performed with the N-terminal peroxidase domain (1-969 amino acids) (MopA-hp) cloned into the pSpeedET expression vector. The expressed protein contains an N-terminal 6× Histidine tag, which was used for NAC purification.

**FIGURE 1:**
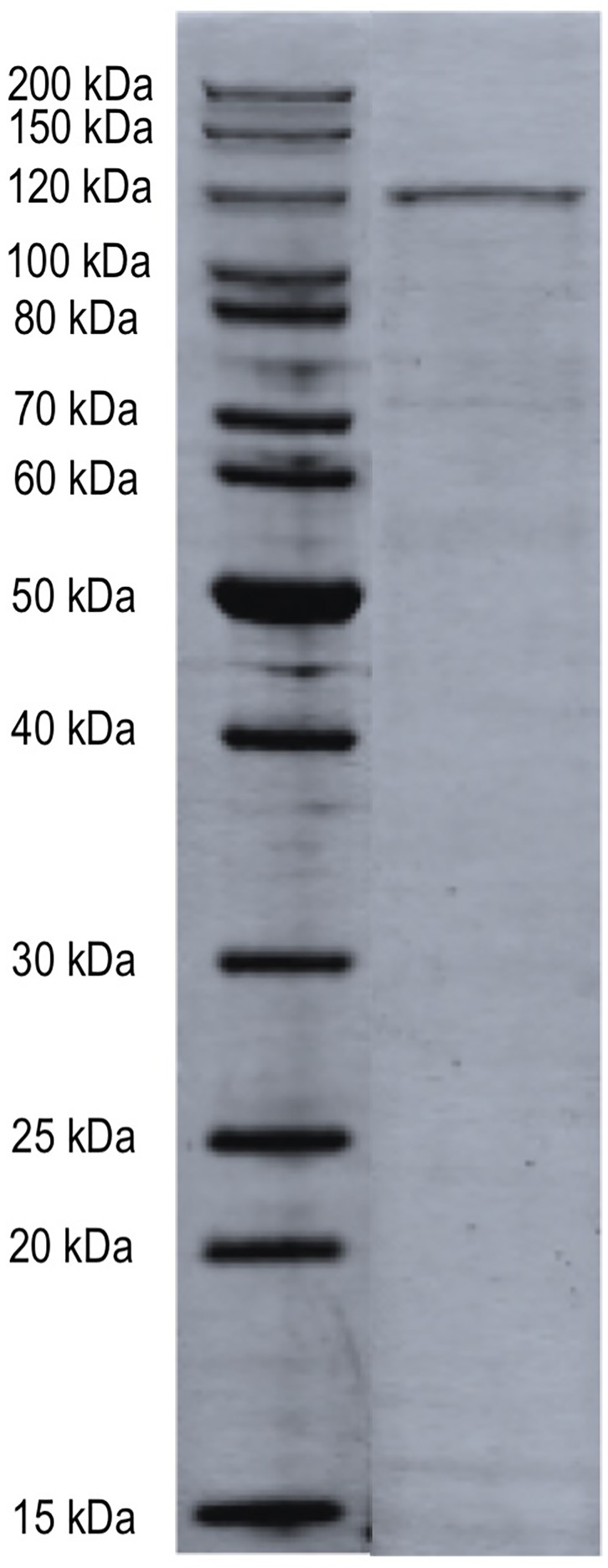
SDS–PAGE of NAC-purified MopA-hp. NAC-purified bulk sample was analyzed by trypsin digestion and LC–MS/MS. The proteins identified in this sample are indicated in Table 3. Molecular weight size standards as indicated.

Nickel affinity chromatography was used to prepare a partially purified MopA-hp. Unfortunately, additional purification techniques led to a complete loss of Mn oxidizing activity or had no impact on purity. Purification of the MopA-hp is indicated in Table 1. Heme, NAD^+^, and calcium were required for activity and PQQ stimulated activity (Table 2 and described below). Mn oxidizing activity was not detected with heat denatured protein or at 4°C. Purification by FPLC or by gravity fed flow led to similar contaminants in the NAC purified MopA-hp, as identified by SDS–PAGE. In order to identify protein contaminants, NAC purified MopA-hp, purified by gravity-driven chromatography, was analyzed by protein LC-MS/MS. The bulk sample clearly identified MopA-hp as the most abundant protein (Figure 1 and Table 3). The emPAI value (Ishihama et al., 2005), which is related to the number of observed peptides vs. the number of observable peptides for a given protein, was determined for each protein identified. This analysis takes into consideration the fact that large proteins, such as MopA-hp, produce more trypsin fragments than small proteins when present at similar levels, but similar to the number of peptides, should not be used for quantification. This analysis likely underestimates the fraction of MopA-hp since large fragments were identified following the trypsin digestion, indicating the trypsin digestion was incomplete. The 10 contaminating proteins with the highest emPAI values are indicated in Table 3. The proteins identified from the *E. coli* expression strain are not likely to be involved in Mn oxidation and have not been identified in other preparations of Mn oxidizing heme peroxidases (Anderson et al., 2009; Andeer et al., 2015; Geszvain et al., 2016). Several of the faint bands are also present in Rosetta^TM^ 2 “purified” protein controls from cells not containing the expression vector (*E. coli* not expressing MopA-hp and “purified” using the same protocol as MopA-hp). The NAC purified MopA-hp was used for further characterization.

**Table 1:** Purification of MopA-hp.

	Volume (ml)	Total protein (mg)	Total activity (nmol min^−1^)	Specific activity (nmol min^−1^ mg^−1^)	Yield (%)	Purification (fold)
Cell-free extract	24.00	100.3	19.3	0.192	100	1.0
NAC	1.45	1.58	1.12	0.709	5.8	3.7

**Table 2:** NAC purified MopA-hp Activity.

Condition	% Activity
Normal activity assay (HEPES buffer, +Ca, +NAD^+^, +heme, +PQQ)	100
Protein prepared and assayed under anaerobic conditions	0
Protein assay prepared aerobically and then purged with nitrogen and assayed under anaerobic conditions	2.0 ± 0.7
+1 mM DMTU in assay	79 ± 3
+SOD (5 μM) in assay	81 ± 23
Absence of PQQ in assay	66 ± 5
Absence of PQQ, +SOD (5 μM) in assay	78 ± 11^a^
TRIS buffered assay	22 ± 2
Assay in dark	97 ± 3
Assay at 37°C	49 ± 2
+25 mM magnesium chloride in assay	32 ± 5

*±standard deviation from minimum of triplicates. ^a^Normalized to assay in the absence of PQQ.*

**Table 3:** Proteins identified in the NAC purified MopA-hp preparation by trypsin digestion and LC-MS/MS.

Proteins identified	Accession number	MW (kDa)	emPAI	Total spectral counts
MopA-hp		105	253	497
Glucosamine-6-phosphate synthase	gi| 118138642	67	9.4	99
Chaperone	gi| 446681610	18	7.78	20
Catabolite gene activator protein	gi| 2098296	24	6.97	21
Dihydrolipoamide dehydrogenase	gi| 446024629	51	5.24	49
Universal stress protein G	gi| 446200654	16	4.84	12
30S ribosomal protein	gi| 446862864	18	4.66	13
Alkyl hydroperoxide reductase subunit C	gi| 445974941	21	4.46	16
DnaK	gi| 446438274	69	4.4	67
Dihydrolipoamide succinyl transferase	gi| 446021967	44	4.09	37
Ferric uptake regulator	gi| 446053853	17	3.85	40

Kinetic parameters were determined for the NAC purified MopA-hp, with a *V*_max_ = 1.08 ± 0.10 nmol min^−1^ mg^−1^, *K*_M_ for Mn of 154 ± 46 μM, and *k*_cat_ = 1.6 min^−1^. Mn concentrations greater than 1 mM inhibited Mn oxidizing activity.

Peroxidase cyclooxygenase enzymes homologous to MopA-hp, such as myeloperoxidase, can catalyze oxidation of chloride with hydrogen peroxide to produce hypochlorous acid (HOCl; Kettle and Winterbourn, 1994). HOCl is able to oxidize Mn(II) abiotically. Although no hydrogen peroxide was added to the assay, potential chloride oxidation resulting in Mn(II) oxidation was investigated. MopA-hp was purified and assayed in chloride-free solutions to prevent potential HOCl formation. When prepared and assayed under these conditions, Mn oxidizing activity was not affected (0.758 ± 0.124 nmol Mn(II) min^−1^ mg protein^−1^ in the absence of added chloride and 0.864 ± 0.040 nmol Mn(II) min^−1^ mg protein^−1^ in the presence of chloride). Potential chloride contamination in the chemical salts used was not determined.

### MopA-hp Is a Hemoprotein

**FIGURE 2:**
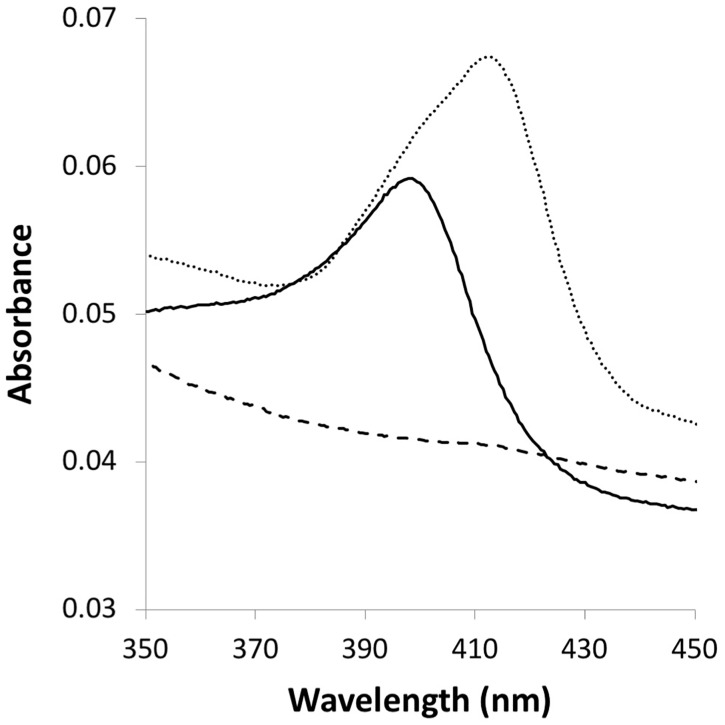
Heme interaction with MopA-hp. A maximum absorbance shift (from 398 to 412.5 nm) within the Soret region results from protein–heme binding. Dashed line: 3 μM NAC purified MopA-hp; solid line: 0.3 μM heme: dotted line: 3 μM NAC purified MopA-hp with the addition of 0.3 μM heme.

Because MopA-hp is similar to heme peroxidases and heme was required for activity, the concentration dependence was investigated. Molar ratios of 0.25–4 of heme: total protein (assuming all protein is MopA-hp, see section “Materials and Methods”) were tested for activity. A heme: protein ratio of 1 would be expected for an active purified protein, but a heme: total protein ratio of 0.25 was sufficient for maximum Mn oxidation activity with NAC purified MopA-hp, which may suggest that “active” MopA-hp is less than a quarter of the total protein present in the partial purification. Binding of heme to MopA-hp was determined spectrophotometrically. An absorbance shift in the Soret region occurs when the protein is added to heme (Figure 2), indicating heme-protein binding.

### Calcium Is Required for Activity

Calcium was previously found to stimulate activity in the native host (Johnson and Tebo, 2008) and was required for Mn oxidizing activity with both the full length and with the heme peroxidase domain when heterologously expressed (Nakama et al., 2014). Similarly, calcium was required for activity with the NAC purified MopA-hp. Mn oxidizing activity increased with calcium concentrations up to 2.5 mM, but did not further increase with up to 40 mM calcium added to the Mn oxidizing assay. Magnesium was unable to replace calcium as a divalent stimulating metal. In fact, addition of 25 mM magnesium sulfate to the assay inhibited activity by approximately 30%. The magnesium could be a competitive inhibitor for Mn(II) or calcium binding to MopA-hp.

### NAD^+^ Is Required for Activity

**FIGURE 3:**
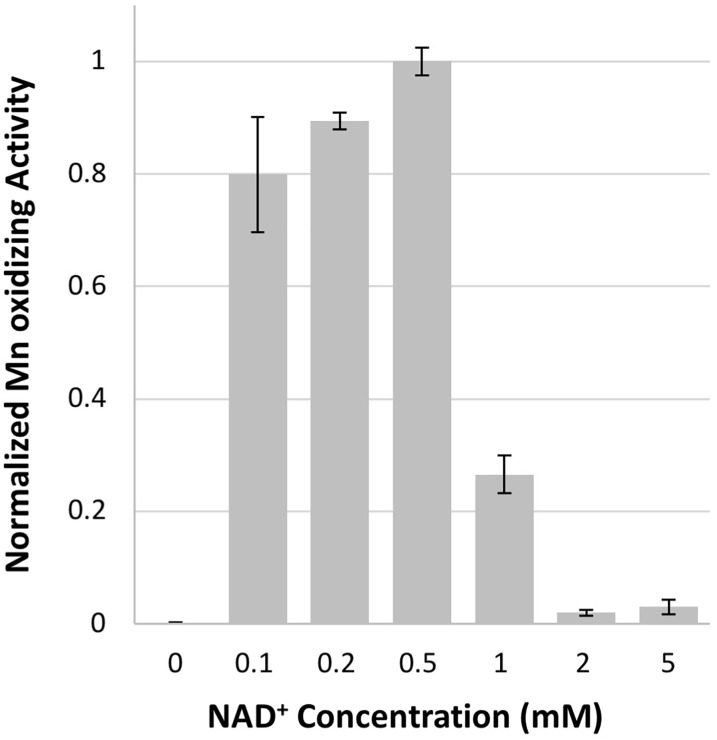
Mn oxidizing activity is dependent upon NAD^+^ concentration. Activity normalized to 0.5 mM NAD^+^ concentration. Error bars represent ± one standard deviation of Mn oxidation assays run in triplicates.

Both NAD^+^ and NADH stimulated Mn oxidizing activity in native *Erythrobacter* sp. SD21 cell extracts (Johnson and Tebo, 2008), but previously showed no effect on activity in *E. coli* cell-free extracts containing the heterologously expressed full length MopA or the heme peroxidase domain (Nakama et al., 2014). In addition, NADH stimulates Mn oxidizing activity with *Roseobacter* sp. AzwK-3b cell-free filtrates (Learman et al., 2011; Andeer et al., 2015). Therefore, the effect of 0.5 mM NAD^+^, NADH, NADP^+^, and NADPH were evaluated for their effect on activity. Only the addition of NAD^+^ resulted in Mn oxidizing activity, and concentrations of NAD^+^ greater than 1 mM inhibited activity (Figure 3). At 0.1 mM, NADH could substitute for NAD^+^, likely because it is rapidly oxidized in the assay (see below).

**FIGURE 4:**
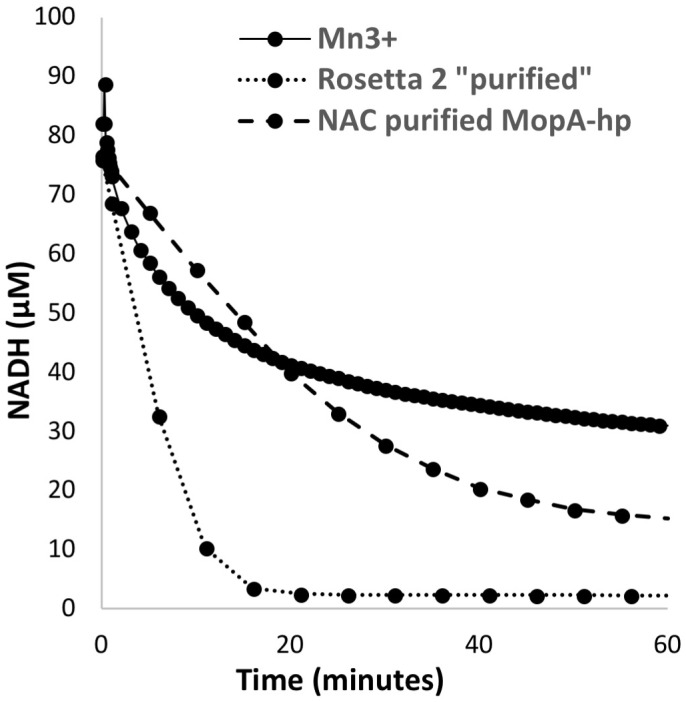
NADH oxidation by 200 μM Mn(III), *E. coli* Rosetta^TM^ 2 “purified” protein (0.11 mg/ml), and NAC purified MopA-hp (0.19 mg/ml).

Despite the unfavorable redox potential of NAD^+^/NADH (*E*′= −0.32 V vs. *E*′ = 1.5 V vs. NHE for Mn(III)_aq_/Mn(II)_aq_), NAD^+^ is a common electron acceptor and might serve as an electron acceptor in Mn oxidation if the enzyme or a ligand can significantly alter the redox potential of Mn(III)/Mn(II), as illustrated with the carbonate complexes of Mn, which can reduce the potential to 0.52 V (Kozlov et al., 2004). The formation of NADH was investigated in the Mn oxidizing assay by monitoring the absorbance at 340 nm. Because of interference at this wavelength from Mn oxides formed during the assay, samples were filtered prior to measurement. No increase in A_340_ was detected when monitored over 24 h. Because Mn(III) and Mn(IV) are strong oxidants, the abiotic oxidation of NADH by Mn(III) was also investigated. As shown in Figure 4, NADH absorbance decreases in the presence of Mn(III) acetate, indicating that NADH is oxidized by Mn(III). In addition, NAC purified MopA-hp (containing identified contaminants) was able to catalyze NADH oxidation (11.15 nmol × min^−1^ × mg^−1^), and Mn was not required for this reaction (Figure 4). Heme and calcium, which are required for Mn(II) oxidation, were not required for NADH oxidation. Rosetta^TM^ 2 “purified” protein controls from cells not containing the expression vector also oxidized NADH at even higher rates (56.3 nmol × min^−1^ × mg^−1^). Thus, it is likely that an *E. coli* Rosetta^TM^ 2 protein contaminating the NAC purified MopA-hp is responsible for NADH oxidation. Because two activities, both enzymatically driven and abiotic, were present to oxidize NADH in the Mn(II) oxidation assay, we used an additional coupled assay to detect NADH formation during Mn(II) oxidation. Pyruvate and lactate dehydrogenase were added to the Mn(II) oxidation assay. Therefore, if NADH was present, lactate would be formed. Because of competing reactions using NADH and the reversibility of lactate dehydrogenase, we would not expect lactate formation to be a quantitative indicator of NADH formation but a qualitative indicator that NADH was formed during the assay. After 24 h of incubation, lactate was determined. Lactate formation, as a proxy for NADH formation, in the Mn-oxidation assay was not above background Rosetta^TM^ 2 “purified” protein levels. Because we were unable to detect NAD^+^ reduction, we investigated whether NAD^+^ may serve as a ligand for Mn(II) but we were also unable to detect an interaction between NAD^+^ and Mn(II) using absorbance spectroscopy or chromatography.

### PQQ Stimulates Mn Oxidizing Activity

Although the mechanism of stimulation is unclear, PQQ has been shown to stimulate Mn oxidation in the native host (Johnson and Tebo, 2008) and in cell-free extracts containing heterologously expressed MopA or the heme peroxidase domain (Nakama et al., 2014). The NAC purified MopA-hp showed a similar response to PQQ addition. The stimulation by PQQ observed with NAC purified MopA-hp (Table 2) is similar to what was observed with cell-free extracts from the heterologous expression. Stimulation of Mn oxidizing activity increases with PQQ concentration up to 10 μM. Higher concentrations of PQQ inhibit Mn oxidation. PQQ may also act as a ligand as the absorbance spectrum of PQQ is shifted in the presence of Mn(II). The absorbance peaks of PQQ at 248 and 339 nm are shifted to 252 and 348 nm, respectively (data not shown).

### The Role of Oxygen and Reactive Oxygen Species in Mn Oxidation

Peroxidases use hydrogen peroxide to oxidize a heme, which can then accept electrons from the substrate, and *Roseobacter* sp. AzwK-3b, which also contains several homologs of MopA, can oxidize Mn by production of superoxide (Andeer et al., 2015). Therefore, special attention was given to the potential role that oxygen, hydrogen peroxide, and superoxide play in MopA-hp catalyzed Mn oxidation. First, the role of oxygen was determined. Oxygen was required for Mn oxidizing activity (Table 2).

**FIGURE 5:**
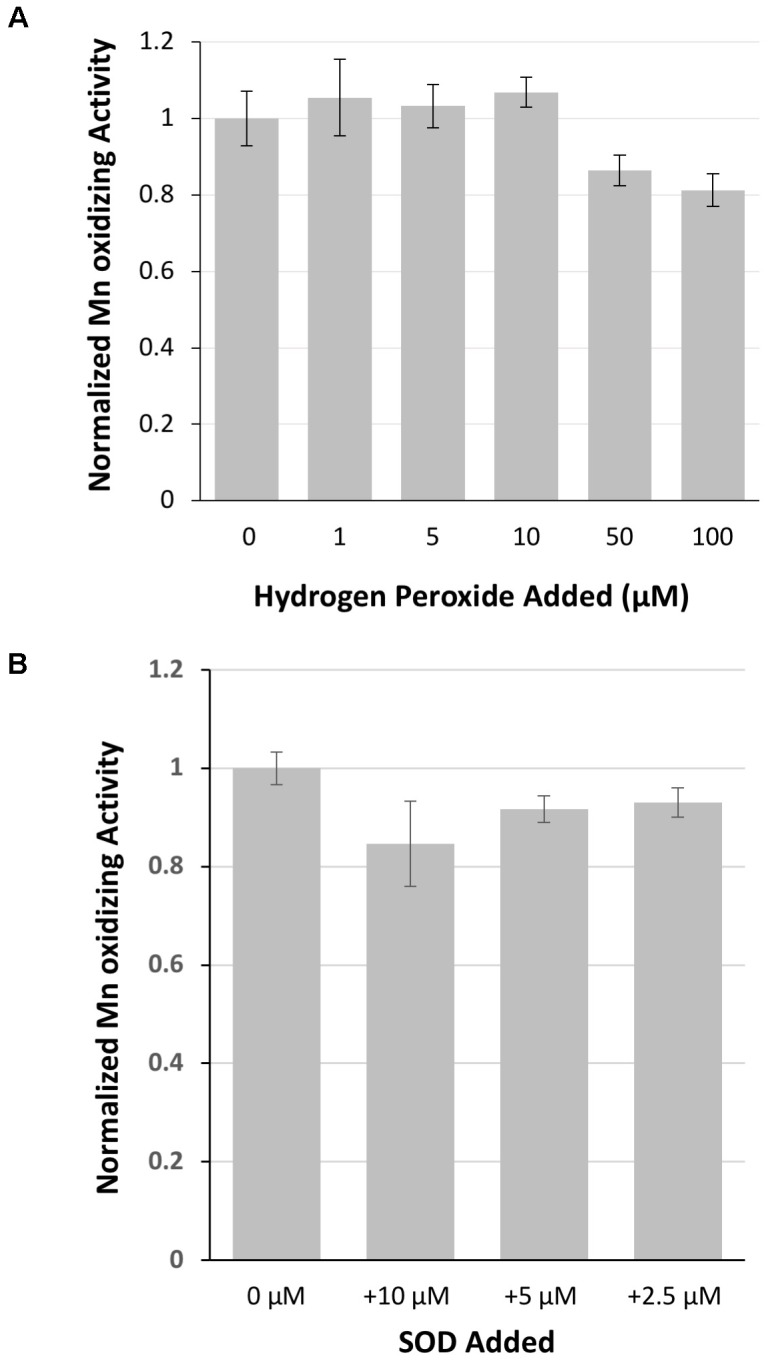
The role of hydrogen peroxide and superoxide on MopA-hp catalyzed Mn(II) oxidation. **(A)** Hydrogen peroxide addition did not stimulate Mn(II) oxidation, and high concentrations of hydrogen peroxide inhibited activity. Activity normalized to no hydrogen peroxide added. **(B)** Superoxide dismutase (SOD), up to 10 μM, has little effect on Mn oxidation. Activity normalized to no SOD added. Error bars represent ± one standard deviation of Mn oxidation assays run in triplicate.

Because of sequence similarity to peroxidases, hydrogen peroxide was investigated as a potential substrate of MopA-hp. Concentrations up to 10 μM had no effect on activity, but 50 μM hydrogen peroxide and greater in the assay inhibited Mn oxidation (Figure 5), likely by reducing oxidized Mn. Catalase, a commonly used hydrogen peroxide scavenger, did not have consistent effects on the assay (data not shown). If heme was not freshly prepared, catalase could stimulate Mn oxidation, suggesting that catalase could donate heme to MopA-hp. Because of this complication with catalase as a potential source of heme, DMTU, a hydrogen peroxide scavenger, was used to identify if hydrogen peroxide was a substrate. When 1 mM DMTU was added to the MopA-hp enzyme assay, Mn oxidation was not significantly inhibited (Table 2). Combined, these data do not support a requirement for hydrogen peroxide to oxidize Mn(II).

Superoxide has been hypothesized to be produced from the MopA-like enzyme of *Roseobacter* sp. AzwK-3b in a reaction using NADH (Andeer et al., 2015). Therefore, the role of superoxide in the *Erythrobacter* sp. SD21 MopA-hp Mn oxidizing activity was also examined by the addition of SOD to the assay. SOD effects on the assay were highly variable between different protein preparations, as reflected in the large standard deviation from 16 independent protein preparations tested in triplicate. Using freshly prepared SOD, addition to the assay could have no effect on Mn oxidation or inhibit it to varying degrees. Adding significant amounts of SOD did not completely inhibit the enzyme (Figure 5 and Table 2), suggesting superoxide is at most playing a minor role in production of oxidized Mn in the assay. Depending on the concentration of PQQ, PQQ may act as an antioxidant or generate reactive oxygen species. Thus, the effect of SOD was also determined in the absence of PQQ. The addition of SOD to the assay in the absence of PQQ decreased activity approximately 20%, similar to the effect in the presence of PQQ. HEPES may produce reactive oxygen species (Keynes et al., 2003; Morris et al., 2011) that may impact Mn oxidation. Addition of catalase to the assay in the absence of NAC purified MopA-hp did not result in Mn oxidation, suggesting any HEPES produced superoxide is not oxidizing Mn(II) abiotically in the assay to detectable levels during the timescales used. In addition, dark conditions and TRIS buffer were tested for the effect on Mn oxidation. There was no difference in activity in the light or dark, unlike that observed with the *P. putida* MopA, and Mn oxidizing activity was significantly lower with the TRIS buffer (Table 2), but it did still occur. TRIS buffer is known to complex metal ions (Fischer et al., 1979).

## Discussion

The Mn oxidizing proteins have been historically difficult to study based on a lack of purity and expression. Insight into the mechanism of action of the MnxGEF complex has recently been achieved due to significant strides in the ability to purify the complex and individual components (Butterfield et al., 2013, 2015; Tao et al., 2017b). These studies have indicated that the MnxGEF multicopper oxidase oxidizes Mn by a unique mechanism involving initial activation by Mn (Soldatova et al., 2017b). Similarly, Mn oxidation by peroxidase cyclooxygenases may occur in an unconventional way. Homogenous purification has not been achieved for this type of bacterial Mn oxidizing enzyme, yet experiments using the heterologously expressed NAC purified MopA-hp do provide strong evidence for the interaction of heme with the enzyme, a clear requirement for NAD^+^, and an opportunity to probe the enzyme mechanism in a defined system.

Identifying *E. coli* contaminants in the NAC purified MopA-hp by MS/MS and using “purified” protein from *E. coli* Rosetta^TM^ 2 not carrying the expression plasmid, we were better able to understand background and interfering activities, such as NADH oxidation. One of these “background” proteins, dihydrolipoamide dehydrogenase can oxidize NADH in the presence of molecular oxygen (Argyrou et al., 2003) and may be responsible for some of the NADH oxidation present in the assay. In addition, the alkyl hydroperoxide reductase can remove hydrogen peroxide by being oxidized by it, although additional subunits would be required to reduce the enzyme allowing greater than stoichiometric oxidation by hydrogen peroxide (Poole, 2005; Yamamoto et al., 2008), thus this enzyme may be playing a minor catalase-like role in the assay. There’s little to suggest most of the other contaminating proteins play any specific role in Mn oxidation or the enzyme assay, especially since similar proteins have not been identified in other MS/MS studies of MopA proteins (Anderson et al., 2009; Andeer et al., 2015; Geszvain et al., 2016). Several of these proteins are chaperones and ribosomal proteins, which may indicate stalled or incomplete protein expression of MopA-hp when using this heterologous system. These proteins may simply interact with MopA-hp to provide a crowded molecular environment that helps to maintain an active protein, such as what was observed during purification of the native protein when addition of protein to chromatography buffers helped maintain activity (Johnson and Tebo, 2008).

Because *K*_M_ values can inform us of the relevance of an enzyme in a given environment, it is interesting to note that the K_M_ for Mn(II) determined for NAC purified MopA-hp (154 ± 46 μM) is quite similar to the *K*_half_ of the Mnx complex of *Bacillus* sp. PL-12, 116 μM (Butterfield and Tebo, 2017), suggesting these enzymes are tuned to environments with similar concentrations of Mn as both strains were isolated near San Diego, CA (Francis et al., 2001; Francis and Tebo, 2002). This is in stark contrast to the much higher *K*_M_ values reported for other multicopper oxidase proteins reported to oxidize Mn(II) such as CotA (Su et al., 2013) and CueO (Su et al., 2014), which have *K*_M_ values of 14.85 and 17.33 mM, respectively. The *K*_M_ value for NAC purified MopA-hp is also much lower than that reported for the MopA-hp domain in cell-free extract (Nakama et al., 2014), which is not surprising since this is a less complex preparation of the enzyme, but it should be noted that these two constructs are slightly different as the previously reported heme peroxidase domain construct was shorter and had lower activity than the construct reported here.

Similar to cell-free extract studies on this protein (Nakama et al., 2014), calcium was required for activity. Although MopA-hp does not contain the calcium binding hemolysin domain, it contains three PERCAL calcium binding motifs (Santamaria-Hernando et al., 2012). Site directed mutagenesis could be used to probe the requirement of all of these sites for activity, but the requirement for calcium is in contrast to the PERCAL containing PepA from *Pseudomonas putida* KT2440 where peroxidase activity was not dependent upon calcium binding (Santamaria-Hernando et al., 2012). In addition, the *K*_D_ for calcium was 12 μM for PepA, which was much lower than the calcium required for full activity with NAC purified MopA-hp. Lactoperoxidase (LPO) is the most similar protein to MopA that has been structurally solved. It contains a calcium ion coordinated by oxygens from Asp110, Thr184, Phe186, Asp188, and Ser190 (Singh et al., 2008). A sequence alignment with MopA indicates that only three of the five residues are conserved, although it should be noted that the much longer length of MopA-hp leads to many sequence insertions throughout the length of the protein when compared to LPO, including around the potential calcium binding site. The high calcium requirement for Mn oxidizing activity may be to fulfill calcium binding at this site. Calcium also stimulated activity with MopA from *Aurantimonas manganoxydans* SI85-9A1 (Anderson et al., 2009).

The covalent heme binding residues identified in LPO, Glu258, and Asp108 (Singh et al., 2008) are conserved in MopA-hp, thus it is not surprising that heme is also required for activity and interacts with the protein. The methionine residue the makes heme binding unique in myeloperoxidase and likely plays a role in catalyzing reactions with high redox potentials (Battistuzzi et al., 2011) does not appear to be conserved in MopA-hp.

The role of NAD^+^, PQQ, and reactive oxygen species are the most intriguing findings as these cofactors would be unusual for a heme peroxidase, and peroxide is expected to be a substrate. Because NAD^+^ is required for activity, and NADH is oxidized rapidly to NAD^+^ by both Mn(III) and contaminants in the NAC purified MopA-hp, NAD^+^ is likely the required cofactor. Studies on the natively expressed MopA had indicated a role for NAD^+^ or NADH in Mn oxidation as both stimulated activity, but neither oxidation nor reduction could be identified (Johnson and Tebo, 2008). The requirement of NAD^+^ for Mn(II) oxidizing activity with NAC purified MopA-hp is consistent with the inhibition of MopA by NADH in *Pseudomonas putida* GB-1 (Geszvain et al., 2016), but also in contrast to findings with *Roseobacter* sp. Azwk-3b where NAD^+^ addition to cell-free filtrates did not affect activity, but activity was stimulated by NADH (Learman et al., 2011). With *Roseobacter* sp. Azwk-3b, NADH is suspected to play a role in superoxide production by MopA which drives Mn(II) oxidation. This is unlikely to be the case here as the rate of NADH oxidation occurs much more rapidly than Mn(II) oxidation. If the two activities were linked through superoxide, one would expect a very rapid initial rate of Mn(II) oxidation, which we do not detect, even in the presence of catalase. NAD^+^ could serve as a ligand of Mn. This would not be unprecedented as NAD^+^ has been shown to bind divalent metals such Cu(II) (Hoffmann et al., 2012) and Mn(II) interacts with the nicotinamide ring of NAD^+^ in the active site of glycosidases (Varrot et al., 2005). Yet, using absorbance spectrophotometry and chromatography, we found no evidence for an interaction between NAD^+^ and Mn.

Pyrroloquinoline quinone is not required for Mn oxidizing activity of NAC purified MopA-hp, but stimulates activity. It is not likely involved in producing superoxide as SOD had similar effects on oxidation in the presence and absence of PQQ. PQQ could serve as a ligand for Mn, and the absorbance shift in the presence of Mn(II) would be consistent with this role. PQQ was previously detected in the partially purified MopA fraction from natively expressed protein (Johnson and Tebo, 2008).

A goal in understanding enzyme function and the potential role of NAD^+^, and PQQ is also to shed light on physiology. The physiological function of Mn oxidation is not well-defined, especially in *Erythrobacter* sp. SD-21. Previous studies on *Erythrobacter* sp. SD-21 have shown that MopA is a soluble cell-associated enzyme likely localized to the cytoplasm or periplasm, or even be a peripheral membrane protein (Johnson and Tebo, 2008), but MopA catalyzed activity has also been identified in cell-free filtrate (Anderson et al., 2009; Sutherland et al., 2018). A localization of MopA outside of the cell seems at odds with a physiological role for NAD^+^. Wood degrading fungi have been shown to excrete NAD^+^/NADH (Kido et al., 2015), but we are unaware of any similar studies demonstrating bacteria secrete the dinucleotide, although extracellular bacterial enzymes requiring NAD^+^/NADH have been described (Lee et al., 1995; Diaz et al., 2013). If intracellular NAD^+^ is involved in electron transfer, quinones could transfer electrons from a loosely membrane bound or periplasmic MopA to intracellular NAD^+^ [as suggested for extracellular NADH stimulated superoxide production (Diaz et al., 2013)], and PQQ may be serving a similar role in the assay, resulting in its stimulatory effect. If NADH is formed, that also would suggest an avenue for energy conservation from Mn(II) oxidation, consistent with increased growth of *Erythrobacter* sp. SD-21in the presence of Mn(II) (Francis et al., 2001). Alternatively, NAD^+^ and PQQ may be serving as viable substitutes for natural electron acceptors or ligands.

Oxygen is clearly required for NAC purified MopA-hp Mn(II) oxidizing activity and it is likely the terminal electron acceptor in this reaction. The lack of stimulation by hydrogen peroxide indicates that hydrogen peroxide is not the oxidizing agent. PepA, a homologous bacterial peroxidase cyclooxygenase, that has been tested for peroxidase activity had a very high *K*_M_ (13 mM) for H_2_O_2_ (Santamaria-Hernando et al., 2012), suggesting these bacterial enzymes may not be very effective peroxidases. In addition, although some superoxide may be produced in the assay and account for a small proportion of Mn oxidized, superoxide is not likely to play a direct role in Mn oxidation with MopA-hp as even 10 μM (>950 U/ml) of SOD did not significantly inhibit activity. This is in contrast to experiments with *Roseobacter* sp. AzwK-3b, where only >50 U/ml could completely inhibit activity with cell-free culture filtrates (Andeer et al., 2015). NADH also stimulated Mn oxidation and superoxide production in *Roseobacter* sp. AZwK-3b cell-free filtrates (Andeer et al., 2015), but with *Erythrobacter* sp. SD-21, NAD^+^ was required and there was no correlation between NADH oxidation rate and Mn oxidation. SOD is also known to have nucleolytic activity (Jiang et al., 2007) with Mn acting as a very efficient cofactor. This activity could potentially be hydrolyzing the dinucleotide bond of NAD^+^, inhibiting the reaction.

The differences in Mn oxidation activity between *Erythrobacter* sp. SD-21 and *Roseobacter* sp. AzwK-3b, despite being catalyzed by MopA type enzymes, may seem odd. But, it should be noted that *Roseobacter* sp. AzwK-3b contains three *mopA*-like genes of varying length, which could indicate different activities for the three different proteins. In addition, the amino acid sequence identity between the *Roseobacter* sp. AzwK-3b MopAs and *Erythrobacter* sp. SD-21’s MopA is approximately 30%, which can be a significant difference for understanding enzyme function and substrate. Recent reports (Sutherland et al., 2018), indicate a superoxide mediated Mn oxidation mechanism in the extracellular fraction of *Erythrobacter* sp. SD-21, as activity is inhibited with 500 U/ml of SOD. This would suggest two potential possibilities – if the protein is actively secreted, secretion involves post-translational modification that alters the activity, or the protein is released into the media as a result of cell lysis and as the protein misfolds and/or degrades, superoxide formation occurs. The latter could also explain the variable results with SOD found here if it is produced when the protein becomes misfolded. It should also be noted that the MopA enzymes are very large (2138 amino acids for the full length *Erythrobacter* sp. SD-21 and 2197 amino acids for the largest subunit found in *Roseobacter* sp. AzwK-3b), and even the heme peroxidase domain is approximately twice as large as the well-studied myeloperoxidases and lactoperoxidases. Therefore, it would not be too surprising that such a large enzyme could have multiple activities that occur in different environments. Despite the differences, it is intriguing to think that in spite of significant divergence in enzyme sequence and mechanism, the ability to produce oxidized Mn remains, which could point to an evolutionary advantage of this process.

Although it remains difficult to develop a mechanistic model of how MopA-hp oxidizes Mn(II) to Mn(III), this study does allow us to rule out potential mechanisms. MopA-hp is not a likely a peroxidase (2Mn^2+^ + H_2_O_2_ + 2H^+^ → 2Mn^3+^ + 2H_2_O) as peroxide addition is not required, and hydrogen peroxide scavengers had little effect on activity. MopA-hp does not likely produce superoxide (NADH + H^+^ + 2O_2_ → ${2O}_{2}^{-}$ + 2H^+^ + NAD^+^), which could then abiotically oxidize Mn(II), as NADH oxidation rates do not correlate with Mn(II) oxidation rates and SOD had little effect on activity. MopA-hp does not likely produce superoxide in the oxidation of Mn(II) with oxygen (O_2_ + Mn^2+^ → Mn^3+^ + $O_{2}^{-}$). If this were the case the superoxide would oxidize Mn(II) to produce Mn(III) and hydrogen peroxide. In the presence of a hydrogen peroxide scavenger, the activity should increase as the hydrogen peroxide would not reduce the Mn(III) formed abiotically back to Mn(II). We did not see an increase in activity in the presence of DMTU. Defining the novel mechanism of MopA catalyzed Mn oxidation can provide important clues to understand the role of Mn oxidation in cellular physiology, the molecular basis of this important biogeochemical reaction, and the vast diversity of enzyme catalyzed reactions.

## Author Contributions

MM, AR, DD, BC, MO, AJ, and HJ designed the experiments and interpreted the results. All authors conducted the experiments. HJ wrote the manuscript with contributions from MM and AR.

## Conflict of Interest Statement

The authors declare that the research was conducted in the absence of any commercial or financial relationships that could be construed as a potential conflict of interest.
